# Research Progress on Data-Driven Industrial Fault Diagnosis Methods

**DOI:** 10.3390/s25092952

**Published:** 2025-05-07

**Authors:** Liang Lei, Weibin Li, Shiwei Zhang, Changyuan Wu, Hongxiang Yu

**Affiliations:** 1 School of Artificial Intelligence, Xidian University, Xi’an 710071, China; 23171214568@stu.xidian.edu.cn (L.L.); wuchangyuan@stu.xidian.edu.cn (C.W.); 2 School of Information Science and Technology, Northwestern University, Xi’an 710127, China; 2022117464@stumail.nwu.edu.cn; 3 Hangzhou Institute of Technology, Xidian University, Hangzhou 311231, China; 24241215273@stu.xidian.edu.cn

**Keywords:** industrial big data, fault diagnosis, deep learning, large language models

## Abstract

With the advent of Industry 5.0, fault diagnosis is playing an increasingly important role in routine equipment maintenance and condition monitoring. From the perspective of industrial big data, this paper systematically reviews the current mainstream industrial fault diagnosis methods. The content covers the main sources of industrial big data, commonly used datasets, and the construction of related platforms. In conjunction with the development of multi-source heterogeneous data, the paper explores the evolutionary path of fault diagnosis methods. Subsequently, it provides an in-depth analysis of data-driven fault diagnosis techniques in industrial applications, with particular emphasis on the pivotal role of deep learning algorithms in fault diagnosis. Next, it discusses the applications and development of large models in the field of fault diagnosis, focusing on their potential to enhance diagnostic intelligence and generalization under big data environments. Finally, the paper looks ahead to the future development of data-driven fault diagnosis methods, pointing out that data quality, interpretability of deep learning, and edge-based large models are important research directions that urgently require breakthroughs.

## 1. Introduction

Since the 21st century, information technology and industrial production have been continuously and deeply integrated. The concepts of Industry 4.0 and Industry 5.0 have been proposed successively, promoting the transformation of industrial production toward digital intelligent manufacturing that respects the Earth’s ecosystem and is human-centered [1]. In the 1970s, the first generation of SCADA (Supervisory Control and Data Acquisition) systems emerged [2]. After half a century of application and development, SCADA systems, in collaboration with various sensors, have been widely applied in industrial production, enabling real-time monitoring of production processes and storing the massive amounts of data generated. Meanwhile, data science has gradually evolved from traditional statistical analysis to integration with artificial intelligence technologies, establishing more precise data models for a deeper understanding and application of the phenomena reflected in the data. The further development and integration of information technology and data science have enabled subsystems in industrial manufacturing to perceive each other, interact in real time, and achieve more effective production models [3].

Industrial equipment is the core component of industrial manufacturing, and its reliability and efficiency are critical factors in maintaining competitiveness within the manufacturing sector. As technology and manufacturing continue to evolve, the structure of industrial equipment becomes increasingly complex, and production processes grow more tightly integrated and interdependent. During equipment operation, undetected or unpredictable faults elevate both maintenance costs and the risk of downtime, ultimately resulting in diminished equipment performance and reduced production efficiency. Depending on the industry, maintenance expenses can account for approximately 15% to 60% of total production costs [4]. Therefore, to ensure the smooth operation of industrial production, conducting fault diagnosis and predicting operational status for running equipment holds significant research value and practical importance.

**Figure 1:**
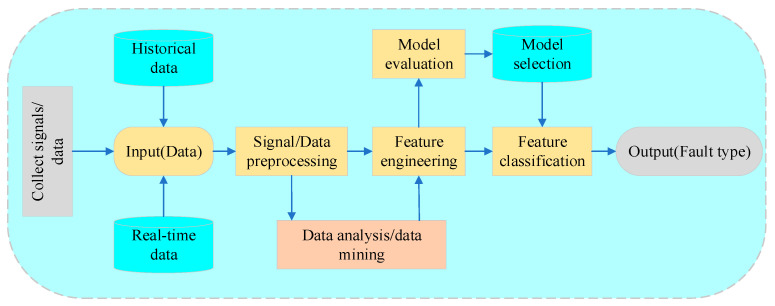
Schematic diagram of fault diagnosis process.

With the comprehensive advancement of industrial digital intelligence transformation, various industrial software and algorithms based on industrial big data have been integrated into industrial products and systems, subsequently giving rise to data-driven fault diagnosis methods for industrial equipment. This has attracted numerous scholars and enterprises to jointly develop fault diagnosis methods suitable for different types of industrial equipment. Figure 1 illustrates the data-driven fault diagnosis process for industrial equipment, primarily encompassing data acquisition and processing, feature extraction, model selection, and feature classification.

Meanwhile, the rapid development of edge computing and cloud computing has made data-driven fault diagnosis methods mainly applicable to two scenarios—embedded monitoring systems and cloud-based maintenance—while also allowing these two approaches to be combined to address complex problems in real-world production. Embedded detection systems deployed at the edge are extensively used in critical scenarios characterized by small data volumes and stringent real-time requirements, such as monitoring and alarming in electrical load applications. When anomalies are detected through power data analysis, the power supply must be cut off immediately, or serious consequences may follow, which greatly safeguards electrical safety and reduces property losses. On the other hand, cloud-based maintenance is primarily aimed at industrial equipment that involves large-scale data analysis and complex predictive maintenance. For instance, General Electric (GE) has developed an industrial internet platform dedicated to connecting, monitoring, and analyzing industrial equipment data, which is widely applied to the predictive maintenance of aircraft engines. Through data analysis, this platform helps airlines detect potential issues at an early stage, thereby optimizing maintenance schedules and reducing operational risks.

The essence of data-driven fault diagnosis lies in collecting the massive volumes of data generated by industrial equipment during operation through sensors and then employing data science and artificial intelligence methods to extract the features embedded in the data, thereby identifying and predicting the equipment’s status to ensure normal operation and optimal performance. Industrial big data provides ample raw data for the development of this field; however, the multi-source heterogeneous nature of industrial data and the explosive growth in data dimensions pose new challenges to data-driven fault diagnosis methods. Therefore, this paper systematically reviews and investigates data-driven fault diagnosis methods, summarizes the existing issues, and proposes research directions that may hold significant importance in the future.

The remainder of this paper is organized as follows: Section 2 discusses big data in the industrial domain, including data sources, data platforms, and their evolution. Section 3 provides a detailed overview of data-driven fault diagnosis methods, highlighting three representative approaches that have made significant contributions to the field, with a focus on their underlying principles and practical applications. In Section 4, representative case studies corresponding to these three approaches are analyzed to provide theoretical support. Section 5 summarizes the current challenges and offers key recommendations for future development. Finally, a conclusion of the paper is presented.

## 2. Industrial Big Data

This section introduces the definition and sources of industrial big data across all scenarios, provides a comprehensive review of existing industrial big data platforms and several commonly used open industrial fault diagnosis datasets, and elaborates on the relationship between the evolution of industrial big data and industrial fault diagnosis methods.

### 2.1. Sources of Industrial Big Data

Industrial big data refers to the entirety of data related to the full lifecycle of products and services in the industrial sector. It encompasses data generated and utilized by industrial enterprises across various stages, such as R&D, production, operation, management, and maintenance services, as well as data from industrial internet platforms [5]. Compared to big data in other fields, industrial big data demonstrates stronger correlations, temporal characteristics, and systemic properties, offering a clearer and more comprehensive reflection of actual production conditions. Moreover, it is well-suited for large-scale collection and storage.

Industrial big data is distributed across each stage of industrial production and typically originates from sensors, the equipment itself, workshop records, business operations, logistics, and so on. With the deep integration of the internet and industry, external internet data has also become an indispensable component of industrial big data. Depending on the scenarios in which data are generated, industrial big data can be categorized into the following three sources:

1. Sensors/Equipment: By installing sensors on equipment, various operating parameters such as temperature, pressure, vibration, and current can be monitored in real time. These data reflect the real-time status of the equipment and can be directly utilized for fault detection and predictive maintenance. Additionally, a large amount of data is generated by the built-in sensors of the equipment itself, which can be directly used for assessing the equipment’s health and swiftly identifying potential issues;
2. Business Operations and Management: Business operations and management data include information such as production planning, business management, commercial strategy, and inventory management. These data typically originate from enterprise management systems like ERP (Enterprise Resource Planning), SCM (Supply Chain Management), and CRM (Customer Relationship Management). Integrating and analyzing these data help enterprises realize refined management, provide new perspectives for decision-makers, and enhance strategic planning;
3. Internet: Internet data is a crucial supplement to industrial big data, especially under the backdrop of widespread internet adoption, which offers more diverse data sources. Such data include user feedback, market demand, weather data, and so on. By combining these external data with internal data, enterprises can carry out more effective product improvements and production planning.

### 2.2. Industrial Datasets and Platforms

In industrial enterprises, the increasing difficulty in acquiring data has led to the incompleteness of fault diagnosis systems [6]. Therefore, the fault diagnosis of each industrial system requires specific datasets. Some scholars and research institutions have collected and developed dedicated fault diagnosis datasets and made them openly accessible. By utilizing these datasets, researchers can test and deploy the diagnostic algorithms they develop. Table 1 lists some of the publicly available and widely used datasets, including their characteristics, data collection locations, and fault types.

Data-driven industrial equipment fault diagnosis primarily utilizes process data collected by sensors/equipment to determine the operational status of industrial equipment and has been preliminarily applied in areas such as industrial software and industrial knowledge management. In August 2015, General Electric introduced Predix [13], the first cloud platform dedicated to industrial big data analytics, providing functionalities including industrial data management, analysis, and cloud computing. As a pioneer among industrial big data platforms, Predix offers strong support for the monitoring and maintenance of industrial equipment. In recent years, Microsoft Azure IoT and Digital Twins [14], combining IoT, cloud computing, and digital twin technologies, have supported the collection and intelligent analysis of industrial big data, furnishing enterprises with a comprehensive solution spanning data acquisition, fault detection, and predictive maintenance.

**Figure 2:**
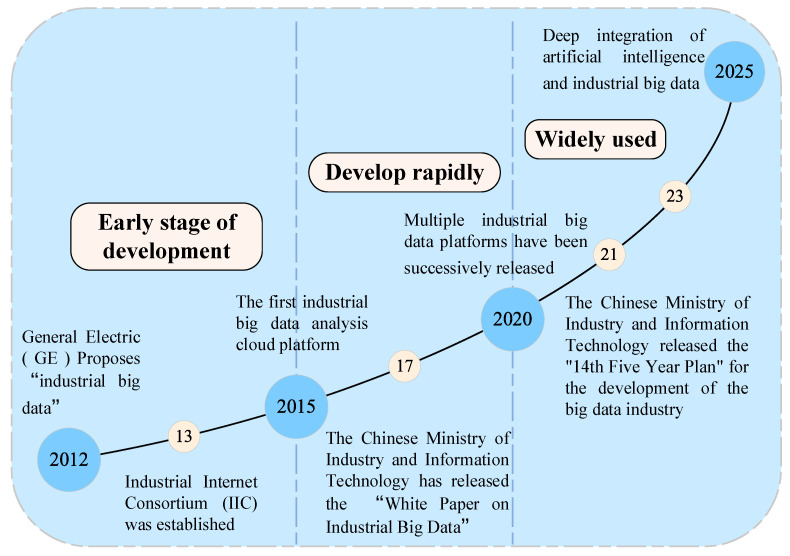
Key events in the development of industrial big data.

In the Chinese market, industrial big data platforms have also achieved significant progress. In 2017, Alibaba launched the “Industrial Brain”, employing cloud computing and artificial intelligence to optimize manufacturing processes and equipment management. In 2018, Huawei released the FusionPlant platform, which supports data collection, processing, and analysis, thus aiding intelligent manufacturing and equipment fault diagnosis. Meanwhile, at its industrial automation forum, ABB announced that its latest industrial IoT platform—ABB Ability [15]—is officially live on Huawei Cloud. The continual updates of industrial big data platforms both at home and abroad highlight the irreplaceable role of data-driven industrial equipment fault diagnosis in the digital intelligence transformation of industry. Figure 2 presents the major milestones in the evolution of industrial big data.

### 2.3. Evolution of Industrial Big Data and Fault Diagnosis

Industrial big data and fault diagnosis methods have evolved in tandem, experiencing three major stages that reflect the mutually reinforcing relationship between technological development and industrial needs. The correspondence across these stages is shown in Table 2.

In the initial stage, due to limitations in data acquisition and storage technologies, the volume of industrial data was relatively small, and the level of automation was low. Fault diagnosis mainly relied on an in-depth understanding of operating principles and expert knowledge. Chao Fu et al. [16] established a relationship between physics-based monitoring variables and the operational status of marine engines, analyzing the underlying mechanisms of different types of faults. They employed a multiphysics model to conduct anomaly detection and fault isolation experiments for two typical faults: lubrication oil filter blockage and cylinder leakage. The results demonstrated that the physics-based model enabled mechanistic reasoning prior to diagnosis, leading to more effective fault separation. The evolutionary characteristics of the model were consistent with the operational patterns of marine engines. Compared with PCA and SAE models, the physics-based approach exhibited superior performance in fault-type discrimination. However, although such methods address equipment maintenance issues to some extent, their ability to identify complex fault patterns is limited. Furthermore, these models lack transferability across different systems, making it difficult to meet the demands for diagnostic accuracy and generalization in modern industrial applications.

As sensor technology and data acquisition systems have advanced, the industrial sector has begun to acquire medium-scale, multi-type signal data, such as vibration, temperature, and pressure. Researchers have started to employ signal processing and analysis models, including time–frequency analysis, wavelet transforms, and Principal Component Analysis, to conduct in-depth examinations of patterns in equipment operation data. Wenjie Zhou et al. [17] applied CEEMDAN and CWT to a dual-disk cracked rotor–rolling bearing system to address the challenge of identifying crack faults under complex vibration signals that may occur in two or more stages of the rotor–bearing operation. CEEMDAN was used to decompose the vibration signals and extract characteristic components specific to the rotor. Subsequently, time–frequency analysis via the CWT spectrogram was employed to validate the accuracy of the decomposition. Experimental results demonstrated that the CEEMDAN–CWT approach can effectively and accurately decompose complex vibration signals. Fault diagnosis methods during this phase improved industrial anomaly detection capabilities; however, challenges remain in handling high-dimensional, multi-source data.

Upon entering the era of big data, industrial equipment has produced exponentially growing volumes of data, which also exhibit increasingly diverse types and dimensions. Faced with large-scale, multi-dimensional, and multi-type data, traditional fault diagnosis methods are no longer adequate. Machine learning and deep learning have become key solutions to this problem. Atma Ram Sahu et al. [4] reviewed the integration of machine learning with fault detection, analyzing historical or real-time data from multiple sources to discover hidden factors behind fault occurrences and pinpoint the exact conditions and timing of system faults. By constructing complex neural network models and training them on large datasets, researchers can extract deep-level features from the data, thereby achieving high-precision equipment fault identification and prediction.

In recent years, the application of pre-trained large models in fault diagnosis has gained extensive attention, leading to the successive introduction of large models for industrial fault diagnosis, such as AnomalyGPT [18] and Myriad [19]. These models boast vast numbers of parameters and powerful feature-learning capabilities, enabling them to capture intricate patterns and relationships from massive datasets and improve both generalization and efficiency. For instance, applying pre-trained large models to the analysis of multimodal data—such as vibration signals, acoustic signals, and images—can yield more accurate detection and prediction of abnormal equipment behaviors.

The success of the aforementioned methods largely hinges on the following factors:

- Data Availability: Whether diagnostic data are accessible or if data can be obtained for a specific component or system;
- Data Types: The known or acquirable data types, such as vibration, acoustic emission, or characteristic signals;
- Data Quality: Whether the data are recorded comprehensively and accurately and contain all the information needed to analyze the features and behaviors of the specific component or system;
- Data Scale: Whether the data volume is sufficient for training fault diagnosis algorithms.

## 3. Data-Driven Fault Diagnosis Methods

Industrial fault diagnosis is vital for ensuring the reliability and service life of equipment across various industries. Generally, it can be classified into three categories: knowledge-based methods, signal processing and analysis model-based methods, and data-driven methods.

1. Knowledge-Based Methods: Knowledge-based methods employ the accumulated experience and expertise of professionals to carry out fault diagnosis. The core principle is to collect and organize expert insights into equipment operation and failure patterns, thereby establishing a set of rules or a knowledge base to guide fault detection and diagnosis. Expert systems and fault tree analysis exemplify this approach [20,21]. These methods are highly interpretable, making the diagnostic process transparent and facilitating comprehension and use by maintenance personnel. Furthermore, they target specific equipment or fault types, offering in-depth analyses. However, they rely heavily on expert knowledge, and gathering and updating that knowledge can be time-consuming and labor-intensive. It may be challenging to cover all potential fault scenarios. As equipment complexity grows, relying solely on expert experience may not meet the requirement for comprehensive diagnosis nor easily adapt to the emergence of new fault patterns;
2. Signal Processing and Analysis Model-Based Methods: These methods utilize various signals produced during equipment operation (e.g., vibration, temperature, acoustic signals) in combination with physical and mathematical models to analyze and diagnose the equipment’s condition. Their fundamental principle is to use signal acquisition and processing techniques to extract fault features, followed by employing analysis models—such as spectral analysis, wavelet transforms, and finite element analysis—to predict equipment performance and possible faults [22]. The advantage of this approach is that it enables real-time monitoring and early warning of failures, providing high diagnostic accuracy for equipment whose physical mechanisms are clearly understood and whose signals are distinct. For instance, vibration signal analysis is widely used for diagnosing faults in rotating machinery. However, these methods demand a high degree of accuracy in the equipment’s physical model and signal quality. Building and validating such models requires specialized domain knowledge, and when confronted with complex, multi-variable systems, models can become overly complex or insufficiently accurate. Additionally, signal noise and environmental interference may reduce diagnostic precision;
3. Data-Driven Methods: Data-driven approaches are increasingly important in preventive maintenance. They collect vast amounts of real-time performance and status data through IoT devices, then perform data fusion and feature extraction, ultimately employing machine learning and deep learning algorithms for fault diagnosis [23]. Such methods exhibit robust adaptability and generalizability, as they can autonomously extract decision-making features from large datasets and handle complex, nonlinear relationships and high-dimensional data without the need for extensive domain expertise upfront—making them well-suited for complex, large-scale industrial datasets. Furthermore, the application of Large Language Models in industrial fault diagnosis achieves end-to-end anomaly detection, enhancing industrial intelligence.

This section primarily provides a comprehensive analysis of data-driven methods, namely traditional machine learning methods, deep learning methods, and modern large-model approaches, including the principles, limitations, and application scenarios of each. Table 3 summarizes and compares the three methods.

### 3.1. Fault Diagnosis Methods Based on Traditional Machine Learning

Traditional machine learning methods principally center on feature engineering and statistical learning theory. Their core objective is to use manual or semi-manual strategies to extract features from raw data that capture both the operational status of equipment and any underlying faults. These features are subsequently fed into conventional machine learning algorithms to build classification or regression models for fault diagnosis.

Feature engineering is pivotal to traditional machine learning, as it directly influences both model performance and generalization [24,25,26]. It primarily comprises feature extraction and feature selection, both of which serve to reduce the dimensionality of data [27,28,29]. Feature extraction entails transforming raw data into representations with strong pattern recognition capabilities, commonly via time-domain, frequency-domain, or time–frequency-domain features. Feature selection involves selecting a subset of features from the original feature set based on specific criteria and can generally be categorized into supervised or unsupervised approaches.

#### 3.1.1. Support Vector Machine

Support Vector Machine (SVM) [30] is a machine learning algorithm for classification, which is most widely used in the field of fault classification. The nuclear technique is used to effectively carry out nonlinear classification, especially for fault diagnosis of rolling bearings, gears, motors, engines, rotor systems, and hydraulic equipment [31,32,33]. The key to SVM achieving accurate classification is the selection of penalty factor and Gaussian kernel function parameters. In order to improve the diagnostic accuracy of SVM-based models, researchers mainly focus on two branches, namely improved SVM and algorithm optimization. Hao Zhang et al. [34] used improved Particle Swarm Optimization (PSO) to optimize the SVM algorithm, optimized the penalty factor of SVM and parameters of Gaussian kernel function through the improved PSO algorithm, and built a PSO-SVM model for fault diagnosis of wind power converters to enhance the accuracy of fault diagnosis.

Hao Zhang et al. [34] adopted an improved PSO to fine-tune the penalty parameter and Gaussian kernel function parameters of SVM, thereby constructing a PSO-SVM model for diagnosing faults in wind power converters and boosting the accuracy of fault diagnosis. Wind power converter fault diagnosis is inherently a nonlinear problem, necessitating the use of a nonlinear mapping that projects data samples into a high-dimensional space for classification. In addition, slack variables $\xi_{i}(\xi_{i}>0)$ are introduced to weight the classification plane, and a penalty parameter *C* is employed to control the penalty imposed on these slack variables. Concurrently, a kernel function $K(x_{i}\cdot x_{j})$ is used to handle the complex decision boundaries in high-dimensional spaces. Consequently, the optimization problem is finally expressed as follows:

(1) $$\underset{w,b,\xi }{\min}\;\frac{1}{2}||w|{|}^{2}+C\sum_{i=1}^{n}\xi_{i}$$

By introducing Lagrange multipliers *α*, the objective function is transformed, converting the optimal hyperplane problem into a dual quadratic programming problem.

(2) $$\underset{\alpha }{\max}\sum_{i=1}^{n}\alpha_{i}-\frac{1}{2}\sum_{i=1}^{n}\sum_{j=1}^{n}\alpha_{i}\alpha_{j}y_{i}y_{j}(x_{i}\cdot x_{j})$$

By solving this problem, the final optimal classification decision function is obtained.

(3) $$f(x)=\mathrm{sgn}\left[\sum_{i=1}^{n}\alpha_{i}y_{i}K(x_{i}\cdot x_{j})+b\right]$$

Experiments indicate that when the radial basis function is employed, the classification performance is optimal, and it can be formulated as follows:

(4) $$K(x_{i}\cdot x_{j})=\exp\left(-\frac{\|x_{i}-x_{j}{\|}^{2}}{2g^{2}}\right)$$

The classification performance of SVM is closely tied to the penalty factor *C* and the kernel function parameter *g*. To select the optimal *C* and *g*, Hao Zhang et al. proposed an improved PSO algorithm. In this approach, a set of particles is randomly initialized, where each particle represents a feasible solution to the optimization problem. The classification accuracy of the SVM fault model serves as the fitness value for each particle. In each generation of the population, there are two extremes: one is the best solution found by the particle itself $p_{best}$, and the other is the best solution found by the entire swarm $g_{best}$. Each particle then moves in the direction of the current optimal particle, and through iterative searches, the global optimum can be reached. Finally, the corresponding SVM parameter combination (C,g) is saved according to the optimal fitness value, and the optimal parameter combination (C,g) and its corresponding classification results are output.

To address the possibility of the algorithm becoming trapped in a local optimum during the later stages of iteration, a crossover operator is introduced to enhance information exchange among particles. The search process is thus jointly managed by individual optimization, group optimization, and individual genetic operations. This compensates for the tendency to get stuck in a local optimum at later stages, enabling the algorithm to escape local optima and achieve a global optimal solution. Experimental results show that, for wind power converter faults, the PSO-SVM diagnostic model demonstrates higher accuracy than traditional SVM models and alleviates the limitations posed by small sample sizes. Nonetheless, it also highlights the shortcomings of conventional machine learning methods in terms of generalization.

**Figure 3:**
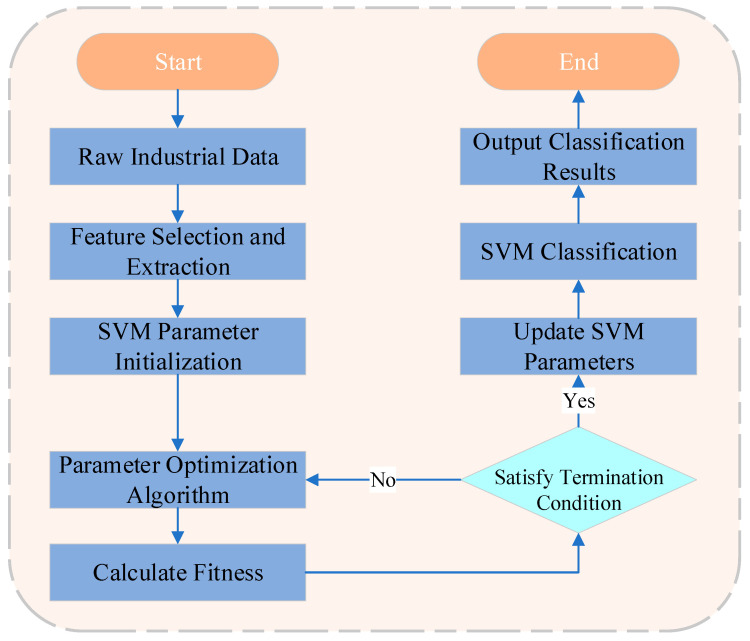
Improved SVM algorithm flowchart.

Furthermore, Zrar Kh. Abdul et al. [35] developed a concat-SVM model based on Mel-Frequency Cepstral Coefficients (MFCC) and Gammatone Cepstral Coefficients (GTCC). They employed two types of global representations—feature concatenation and feature statistics—to feed data into the SVM, conducting experiments on two different datasets, PHM09 and DDS. Their results indicate that the concat-SVM model effectively addresses vibration fault detection in gears and helical gears, outperforming the other models tested. The superior performance of concat-SVM lies in its ability to comprehensively represent vibration signal features. Luigi Russo et al. [36] proposed a One-Class Support Vector Machine (OC-SVM) method for the early detection of anomalies in steel mills, validated using the Pittini dataset. Their analysis showed that the OC-SVM model can be successfully applied for fault diagnosis in the steel industry. Hyunseong Lee et al. [37] introduced an anomaly detection framework based on Support Vector Regression (SVR) for safety monitoring in commercial aircraft. By leveraging FDR datasets, they performed real-time anomaly detection and observed abrupt changes in monitored features; compared to recorded features, anomalies were flagged one to three seconds before the actual occurrence. Such algorithmic optimizations are aimed at refining complex solutions and streamlining the parameter selection process for SVM. These approaches demonstrate superior diagnostic performance compared to traditional SVM-based methods. Figure 3 provides a summary of the workflow for these methods.

SVM-based diagnostic models are trained by minimizing structural risk, which confers a solid theoretical foundation and improves model interpretability. Because the optimization objective is a convex quadratic programming problem, it is feasible to find a global optimum, thereby achieving high diagnostic accuracy. Nevertheless, SVM diagnostic models have three main drawbacks:

1. Scalability: When dealing with massive datasets, computational complexity increases dramatically and can lead to a computational burden of catastrophic proportions;
2. Sensitivity to Kernel Functions: Model performance is highly sensitive to the selection of the kernel function and its parameters. Inappropriate choices can fail to yield reliable diagnostic results;
3. Multi-Class Complexity: Originally designed for binary classification tasks, SVM requires complex strategies—such as one-vs.-rest or one-vs.-one—to integrate multiple SVM models for intelligent fault diagnosis in multi-class scenarios, thus adding to both model complexity and computational overhead.

Hence, when applying SVM to large-scale or multi-class diagnostic tasks, these limitations should be carefully assessed, and additional methods or improved strategies should be considered to overcome these challenges.

#### 3.1.2. Other Machine Learning Models

Beyond the widespread application of SVM algorithms in industrial fault diagnosis, other machine learning models have also garnered significant attention from researchers, including KNN (K-Nearest Neighbors), PGM (probabilistic graphical models), and decision trees. KNN leverages distance metrics to identify the nearest neighbors of unlabeled samples and has been successfully applied in diagnosing faults in rolling bearings [38,39,40], gears [41,42], and motors [43]. PGM represents probabilistic relationships among variables through graphical structures, encompassing Bayesian classifiers and Markov models. Naive Bayes classifiers and hidden Markov models have been utilized to identify the health states of rolling bearings [44,45], gears [46,47], synchronous motors [48], and hydraulic pumps [49]. To further enhance generalization, ensemble methods such as random forests have also been introduced and have been applied to fault diagnosis in induction motors and gearboxes [50].

Nevertheless, these methods face several limitations. KNN exhibits a high computational cost when dealing with large-scale data, making it difficult to define neighborhood boundaries; it also suffers from challenging parameter selection and reduced diagnostic accuracy under imbalanced data distributions. PGM has a weaker capacity for modeling complex functional relationships; model construction becomes difficult if the probabilistic relationships among variables are not clearly defined. Decision trees are prone to overfitting, resulting in poor generalization, and often rely heavily on expert knowledge during model building. These shortcomings restrict their effectiveness in complex fault diagnosis tasks and call for further improvement and optimization.

Traditional machine learning methods have played a pivotal role in industrial equipment fault diagnosis, particularly under conditions of limited data volume and well-defined fault characteristics, where they possess irreplaceable advantages. However, they tend to be insufficient when confronted with large-scale, multi-modal industrial data and are highly sensitive to environmental changes, resulting in weaker generalization and reduced adaptability across different working conditions or equipment. Consequently, such methods no longer satisfy the current requirements of industrial fault diagnosis, prompting research efforts to increasingly shift toward more robust deep neural network approaches.

### 3.2. Deep Learning-Based Fault Diagnosis Methods

In recent years, deep learning methods have found extensive application in industrial fault diagnosis. By leveraging a deep, layered architecture to learn features at multiple levels of abstraction, these methods can directly establish a relationship between the learned features and the target outputs. Since they capture all intermediate features between raw data and higher-level classification [51], deep learning methods exhibit strong generalization capabilities. Commonly used deep learning algorithms include Deep Belief Networks (DBNs) [52], Convolutional Neural Networks (CNNs) [53], Recurrent Neural Networks (RNNs) [54], Generative Adversarial Networks (GANs) [55], Deep Transfer Learning (DTL) [56], and Reinforcement Learning (RL) [57]. These categories encompass a wide range of variants, such as Long Short-Term Memory (LSTM) [58] and Gated Recurrent Units (GRUs) [59], among others.

#### 3.2.1. Deep Belief Network

**Figure 4:**
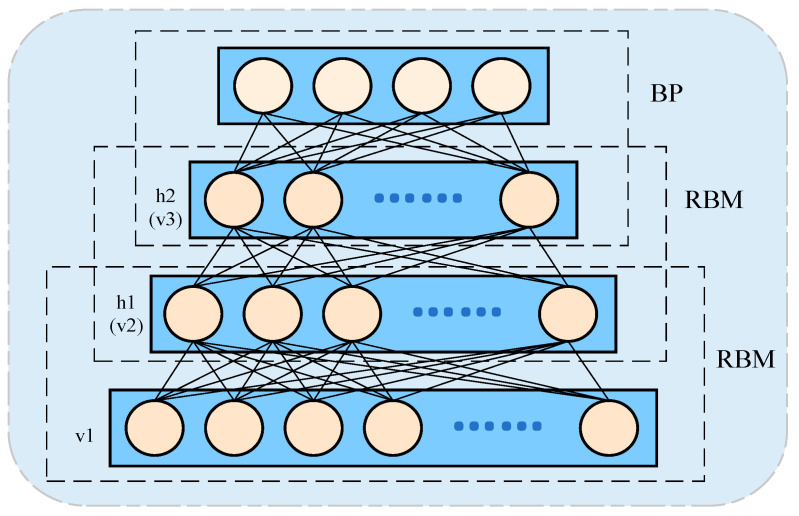
DBN network structure diagram.

Proposed by Hinton et al. in 2009, a Deep Belief Network (DBN) is a hybrid probabilistic generative model. It can be viewed as a combination of multiple Restricted Boltzmann Machines (RBMs) [60]. A commonly used DBN architecture is illustrated in Figure 4, where a BP (BackPropagation) network is typically added in the final layer as a classifier [61]. The hidden layer states of one RBM serve as the visible layer inputs for the next RBM. Unlike traditional machine learning approaches, DBN-based diagnostic models can be trained by pre-training a stack of RBMs to automatically learn high-level features from input data. By directly leveraging raw signals or spectra, these models enable the construction of end-to-end intelligent diagnostic systems, thereby reducing dependence on expert experience and domain knowledge.

A DBN is capable of extracting meaningful fault features from feature sets constructed for specific tasks. Tamilselvan et al. [62] extracted both time-domain and frequency-domain features from collected multivariate time series and applied these features to DBN training, successfully classifying aircraft engine faults. Likewise, Haizhou Chen et al. [63] employed the Intrinsic Mode Functions (IMFs) derived from vibration signals processed by Ensemble Empirical Mode Decomposition (EEMD) to form a low-dimensional feature set, which they then used as DBN input.

To enable DBNs to adapt to more specialized fault diagnosis tasks, hybrid approaches and algorithmic enhancements are often necessary to improve diagnostic reliability. Huixin Tian et al. [64] proposed a new fault diagnosis method based on spatio-temporal features fusion based on deep belief network (STF-DBN), built upon a DBN architecture, targeting the multi-dimensional features of compressor condition-monitoring data by extracting features from both spatial and temporal dimensions. The essence of STF-DBN lies in its feature extraction strategy and fault diagnosis strategy. In feature extraction, vibration, temperature, and pressure signals are comprehensively considered throughout the reciprocating compressor’s operating cycle, taking advantage of the DBN’s strong feature-extraction capabilities to integrate various parameter information and obtain multi-dimensional fault features in both time and space. For fault diagnosis, rather than adopting a traditional fault classification approach, STF-DBN constructs a health curve based on the extracted features to determine the compressor’s operational stage (healthy, fault warning, or fault) and diagnose the fault type. The overall structure of the STF-DBN-based fault diagnosis method consists of an input layer, time-domain feature extraction layer, feature processing layer, weight allocation layer, spatial feature extraction layer, and output layer. Experimental results involving TE process faults, compressor valve faults, and motor faults show that the proposed method’s fault-warning performance significantly exceeds that of Principal Component Analysis (PCA), indicating certain advantages over traditional multivariate statistical process monitoring methods.

Additionally, Zhuyun Chen et al. [65] proposed a Sparse Autoencoder–Deep Belief Network (SAE-DBN) model. By using an SAE to integrate time-domain and frequency-domain features from multiple sensor signals and then applying the fused feature vectors to train a DBN, they achieved the classification of rotating machinery faults. Defeng Lv et al. [66] introduced a semi-supervised fault diagnosis method for aircraft engines, leveraging a Denoising Autoencoder (DAE) together with a DBN. The DAE performs unsupervised learning on faulty samples to accomplish feature denoising and dimensionality reduction, and the extracted features—along with their labels—are then fed into the DBN for supervised learning.

#### 3.2.2. Convolutional Neural Network

**Figure 5:**
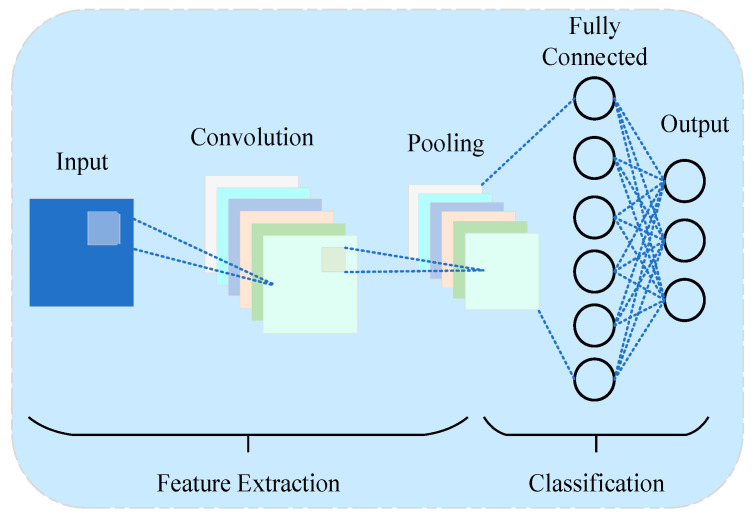
CNN structure diagram.

Convolutional Neural Networks (CNNs) first appeared in the LeNet model proposed by LeCun, specifically designed for data with a grid-like topology. Since then, CNNs have demonstrated remarkable power in image processing, leading to various CNN-based models such as VGGNet [67], GoogleNet [68], and ResNet [69]. A typical CNN architecture consists of three primary components: convolutional layers, pooling layers, and fully connected layers. Its defining features include local receptive fields and parameter sharing. Figure 5 illustrates the main structure of a CNN.

**Figure 6:**
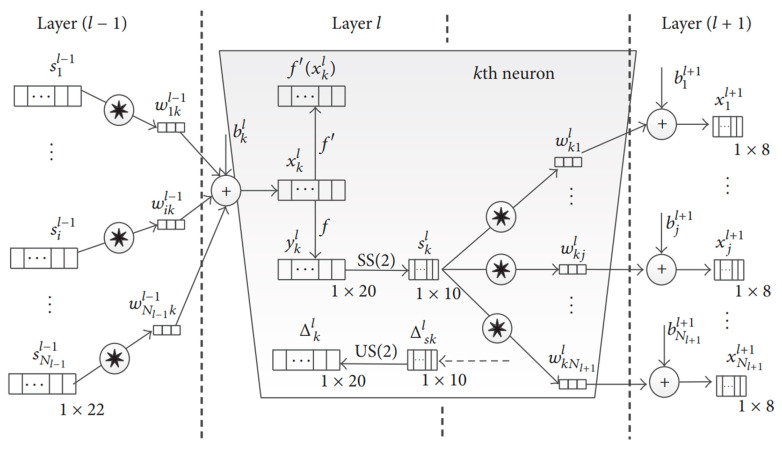
Convolution layers of the proposed adaptive 1D CNN configuration.

By employing local receptive fields and weight sharing, CNNs can handle large-scale data with fewer parameters, which is advantageous for processing high-frequency vibration signals. Ince et al. [70] applied a one-dimensional CNN directly to input signal samples obtained from motor current, enabling rapid and accurate detection of early motor bearing faults. This approach circumvents the manual feature extraction steps typically needed for varying operational conditions. Specifically, the authors used an adaptive one-dimensional CNN for both feature extraction and fault detection from raw motor current signals. The adaptive 1D CNN structure allows for handling input layers of arbitrary dimensions, as illustrated in Figure 6. Furthermore, the proposed compact CNN incorporates hidden neurons in convolutional layers that can perform both convolution and subsampling operations. Because of this fusion, the convolution and subsampling layers are referred to collectively as “CNN layers”, while the remaining layers are still termed “MLP layers”. Consequently, a one-dimensional CNN can be summarized as comprising an input layer, hidden CNN layers, MLP layers, and an output layer. Compared with traditional two-dimensional CNNs, the proposed one-dimensional CNNs exhibit further structural differences. The primary distinction lies in the use of one-dimensional arrays for kernels and feature maps instead of two-dimensional matrices. Consequently, operations such as 2D convolution and lateral rotation are replaced by their one-dimensional counterparts, namely 1D convolution and reverse operations. In one-dimensional CNNs, the kernel size and subsampling parameters are now scalar values. However, the multilayer perceptron (MLP) layers remain consistent with their two-dimensional counterparts, thus preserving the same conventional BP formulations.

CNN has found broad applications in fault diagnosis, with extensive literature available. The following discussion focuses primarily on fault detection methods based on vibration data. Complex operating environments and intricate equipment structures lead to highly complex vibration signal components. To address the challenges arising from these external and internal factors, numerous approaches have been proposed to enhance CNN-extracted features in various ways. For instance, Kuigeng Lin et al. [71] proposed a health management model based on Temporal Convolutional Network (TCN) [72] and Support Vector Data Description (SVDD) [73], employing Hilbert Spectrum Fusion Technology (HSFT) to boost model performance. By establishing a one-class classifier for vibration prediction residuals, they achieved vibration anomaly detection with superior results on both simulated and real monitoring datasets. Zhao M. [74] introduced a ResNet variant with dynamic frequency band weighting to improve the feature extraction capability of CNNs. Since there is no general consensus on which wavelet basis functions are best suited for diagnosis, Zhao M. et al. [75] developed a wavelet coefficient-based fusion method in an attempt to obtain a more comprehensive time-frequency representation of vibration signals, subsequently using the fusion results to train a CNN-based diagnostic model. Jiang G. et al. [76] proposed a Multi-Scale CNN (MSCNN) that extracts multi-scale features through a multi-branch architecture, thereby acquiring complementary and rich diagnostic information—in other words, the “multi-scale features” embedded within vibration signals. Peng D. et al. [77] presented a multi-branch architecture incorporating a denoising branch to extract complementary fault information. Similarly, following the idea of integrating complementary information, Wang H. et al. [78] introduced a multi-branch architecture with a denoising branch to capture complementary fault information by stacking vibration signals from multiple sensors into two-dimensional images for CNN training, thus enabling the extraction of more diverse features. Zhang W. et al. [79] proposed a Training-Interference CNN (TICNN) method in which a wide convolutional kernel is used in the first layer to suppress high-frequency noise, and noise is introduced during training to enhance the model’s noise robustness, thereby improving diagnostic performance in noisy environments. Han Y. et al. [80] introduced an Enhanced CNN (ECNN) that employs dilated convolution to expand the receptive field, comprehensively capturing both short-range and long-range fault information, as well as leveraging long-range dependencies among vibration signals.

Compared to DBNs, CNN-based diagnostic models are capable of directly learning features from raw monitoring data without requiring frequency-domain transformations or other preprocessing steps, thanks to the flexible structure and powerful feature extraction capabilities of CNNs that can capture variations in the input data. To mitigate the adverse effects of nonlinearity and noise interference in vibration signals, various modifications have been made to CNN and its variants, including multi-branch architectures, cascaded structures, attention modules, and dynamic coefficient modules, which collectively enhance the feature extraction capacity to some extent. During the training phase, adversarial training is increasingly used to reduce cross-domain discrepancies, thereby achieving transfer fault diagnosis. However, most studies have yet to prioritize the optimization of training strategies, such as adaptive learning rates and batch normalization.

#### 3.2.3. Recurrent Neural Network

**Figure 7:**
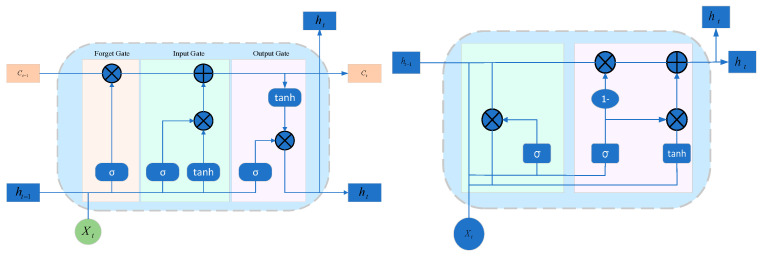
General structure of LSTM and GRU.

Recurrent Neural Networks (RNNs) trace their origins to the Hopfield Neural Network, in which the connections among network nodes form a directed graph over time. This design grants RNNs a unique advantage in handling time-series data. However, due to the long-range dependencies inherent in RNNs, gradient vanishing and exploding gradients can occur during training [81]. Consequently, most RNN-based methods in fault diagnosis heavily rely on improved variants, such as LSTM [82] and GRU [59], as illustrated in Figure 7.

Leveraging the strong time-series modeling capability of RNNs, Chaofan Tang et al. [83] introduced an interpretable multivariate time-series anomaly detection approach called GRN, built on GRU and graph structures (network architecture shown in Figure 7). GRN automatically learns potential correlations among sensors in multi-dimensional industrial control time-series data, thereby capturing both long-term and short-term dependencies to enhance detection performance and aid users in tracing the root causes of detected anomalies. While preserving an RNN’s core strength of processing sequences and modeling temporal relationships, GRN also alleviates the problems of gradient vanishing and explosion. When compared against nine state-of-the-art algorithms on two real-world water treatment datasets (SWaT and WADI), GRN demonstrated superior detection accuracy and recall.

**Figure 8:**
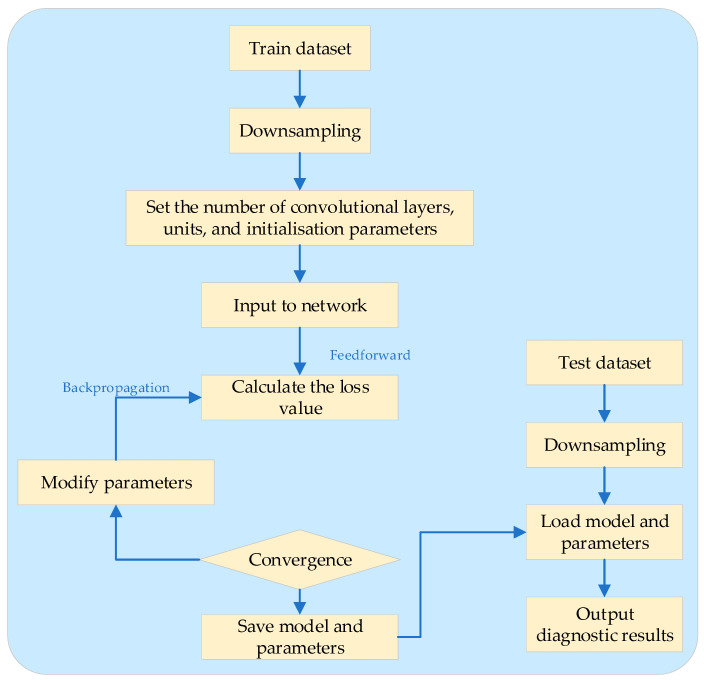
MCNN-LSTM flow block diagram.

To harness the advantages of various deep learning architectures for fault diagnosis, some existing methods integrate different deep learning models with LSTM or GRU. For instance, Chen X. et al. [84] employed a Multiscale Convolutional Neural Network–Long Short-Term Memory (MCNN-LSTM) model for fault diagnosis. This model comprises a feature extractor and a classifier, allowing raw data to be directly input into the model without the need for preprocessing. The process flow is illustrated in Figure 8. The feature extractor consists of two CNN branches with different kernel sizes, designed to automatically extract representative features from the vibration signals of rolling bearing faults. The extracted features are then fed into a stacked LSTM network, which serves as the classifier for fault evaluation. This architecture significantly reduces the number of model parameters while maintaining diagnostic performance. Liu H. et al. [85] proposed a GRU-based Nonlinear Prediction Denoising Autoencoder (GRU-NP-DAE), detecting anomalies and classifying faults using the reconstruction error between the predicted data and the actual data, thereby effectively mitigating noise-related challenges. Recognizing that a CNN-only approach may overlook the temporal relationships in time-series data, Zhang Y. et al. [86] incorporated GRU units into the network architecture to learn feature representations from two-dimensional images of stacked time series, subsequently classifying those features with an MLP. Similarly, Ning S. et al. [87] put forward an improved ShuffleNetV2-LSTM intelligent fault diagnosis system, retaining ShuffleNetV2’s capability for feature extraction while tapping into LSTM’s advantage in modeling sequential data, ultimately enhancing diagnostic accuracy.

Compared with other deep learning algorithms, RNNs are characterized by their ability to handle the dependence on long sequential data, providing a robust solution for industrial fault warnings. In Intelligent Fault Diagnosis, RNN variants are largely based on LSTM and GRU, particularly in multi-layer, bidirectional configurations. Owing to the distinct edge RNNs offer in processing time-series data, numerous contemporary solutions employ these RNN variants or combine them with other classification algorithms, striving to preserve the temporal correlations of vibration signals during feature extraction so as to improve diagnostic performance.

#### 3.2.4. Generative Adversarial Network

**Figure 9:**
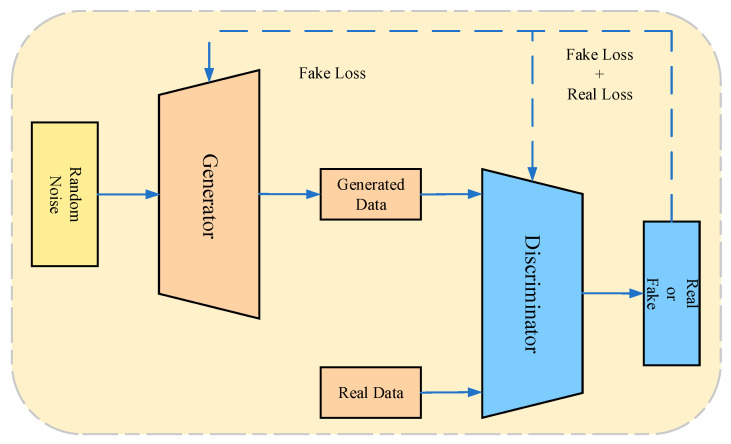
GAN structure diagram.

Generative Adversarial Networks (GANs) were first introduced by Goodfellow et al. in 2014 [88]. As an unsupervised learning algorithm, GANs enable two independent neural networks to train in an adversarial manner. The general GAN architecture includes a generator *G* and a discriminator *D*. The generator *G* aims to capture the input data’s distribution, training *G* to maximize the probability that *D* makes a wrong judgment; the discriminator *D* estimates whether a sample comes from the training data rather than from *G*, training *D* to differentiate whether the sample was generated by *G*. Figure 9 depicts this process. GANs provide an excellent generative model that does not rely on prior knowledge or assumptions about data distribution, instead using maximum likelihood estimation to model the data. In practical applications, numerous GAN variants exist, such as Deep Convolutional GAN (DCGAN), Conditional GAN (CGAN), and Auxiliary Classifier GAN (ACGAN) [89].

**Figure 10:**
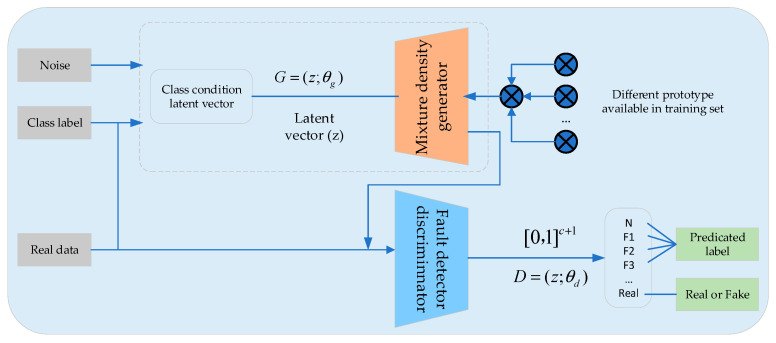
MoGAN architecture diagram.

In many real-world scenarios, data often exhibit a long-tailed distribution, with healthy data typically outnumbering fault data by a large margin. This data imbalance can adversely impact a model’s fault diagnosis capabilities. Consequently, the strong generative ability of GANs has been increasingly employed to address this issue [90]. For example, Zareapoor M. et al. [91] proposed a deep learning model based on the Auxiliary Classifier GAN (ACGAN), referred to as the Minority Oversampling GAN (MoGAN). The detailed architecture of this algorithm is illustrated in Figure 10. In this approach, a conditional latent vector is constructed by combining random noise with embedded fault class labels, which drives a hybrid-density generator to synthesize minority-class vibration/current samples through a multi-prototype convex combination within the neighborhood of the majority-class manifold. The discriminator is designed with k + 1 output channels, integrating binary real/fake discrimination with the multi-class classification of “normal + fault types”, thereby unifying oversampling and diagnosis while enhancing gradient signals for minority classes. Additionally, a Feature-Matching loss is introduced to stabilize training and ensure that the high-dimensional statistical features of generated samples closely resemble those of real fault signals, effectively balancing oversampling quality and diagnostic accuracy. Zhimin Du et al. [92] combined GAN with an incremental learning SVM model to mitigate data imbalance in heating, ventilation, and air conditioning (HVAC) systems, achieving an acceptable level of diagnostic accuracy. Additionally, Li Z. et al. [93] integrated ACGAN with Wasserstein GAN (WGAN) and introduced gradient penalty into the system, creating the ACWGAN-GP model for expanding imbalanced datasets. Experimental results show that this model can produce synthetic samples highly similar to real-world data, thereby improving fault diagnosis accuracy.

Due to the structure of GANs, their inputs typically consist of time-domain, frequency-domain, or time–frequency-domain signals. When dealing with small sample sizes or imbalanced datasets, the generative capability of GANs offers valuable data augmentation for fault diagnosis. Methods such as online filtering or integrating DAEs with GAN improve the quality of generated data, while ACGAN—featuring a built-in fault classifier—can handle fault classification directly. In industrial fault diagnosis, GANs are primarily applied for data generation, and adversarial training has been shown to improve diagnostic accuracy.

#### 3.2.5. Deep Transfer Network

**Figure 11:**
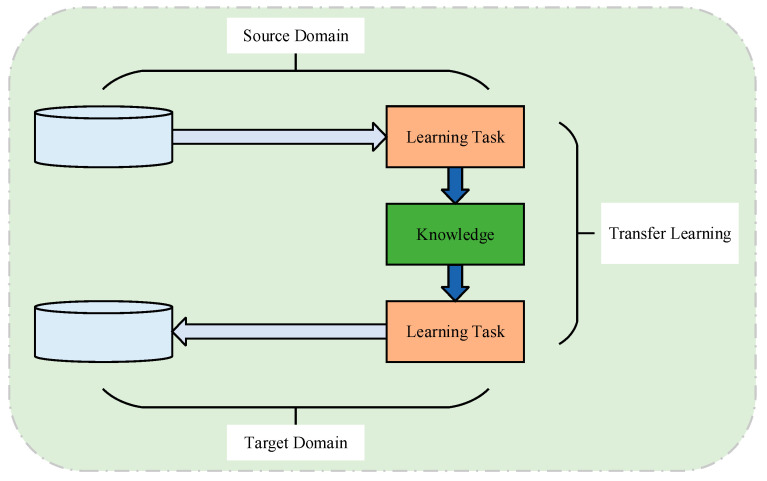
Transfer Learning architecture diagram.

The term “Transfer Learning” (TL) was introduced into the field of machine learning by Lorien Pratt, who developed discriminative transfer algorithms. TL refers to a methodology that facilitates the transfer of knowledge from a previously learned task to the learning of an auxiliary target task [94]. At its core, TL aims to identify similarities between existing and new knowledge, emphasizing the capability to transfer and adapt knowledge across different domains or evolving tasks. Two fundamental concepts in TL are the domain and the task. These are typically denoted as the source domain (SD) and target domain (TD), as well as the source task and target task, respectively, as shown in Figure 11.

Deep learning models typically require large volumes of labeled data to achieve optimal performance. However, in industrial environments, this requirement is often impractical, as collecting such data—particularly fault-related samples—is both time-consuming and challenging. Transfer Learning effectively addresses this limitation, especially in scenarios involving few-shot learning, zero-shot learning, and online fault detection.

Danmin Chen et al. [95] proposed a real-time fault diagnosis method based on a two-tier deep transfer neural network (TTDNN) to address the challenges of high computational complexity and large-scale convolution operations associated with high-dimensional data. This method integrates multi-source heterogeneous information through a two-stage Transfer Learning process, effectively avoiding convolution operations and enabling real-time fault diagnosis. Shi et al. [96] proposed an intelligent bearing fault diagnosis method that combines one-dimensional Convolutional Neural Networks (1D-CNNs) with a Transfer Learning (TL) layer, as illustrated in Figure 8. A Gated Recurrent Unit (GRU) is incorporated to better capture long-term dependencies in time series data, followed by an attention layer to automatically select the most relevant features. The method was validated under small-sample and variable-load conditions, demonstrating superior generalization performance compared to models without the transfer layer. Specifically, it achieved over 99% accuracy when the target domain data ratio was high and maintained above 97% accuracy even under limited target data conditions. Guiting Tang et al. [97], inspired by lightweight network design and envelope demodulation signal processing techniques, introduced a 2D Convolutional Neural Network (2D-CNN)-based Transfer Learning (TL) model for bearing fault diagnosis. This model adaptively adjusts the input length to make the network more lightweight and improve computational efficiency. Evaluated across 81 cross-domain diagnostic tasks, experimental results demonstrated both high diagnostic accuracy and improved lightweight characteristics.

In the field of industrial fault diagnosis, Deep Transfer Learning has emerged as a pivotal solution for addressing challenges related to label scarcity, variable operating conditions, and real-time deployment. It enables rapid adaptation of well-trained models to new equipment, operating conditions, or fault types, even with very limited or completely unlabeled target data. Deep Transfer Learning can be effectively combined with data augmentation techniques—such as GAN-based sample generation—and federated learning to enhance model generalization while ensuring data privacy compliance. Moreover, parameter-efficient fine-tuning techniques have significantly lowered the barrier for deployment on edge devices. However, excessive divergence between source and target domains may lead to negative transfer, and current methods lack a unified framework for trustworthiness assessment and safety verification. Adaptive scheduling mechanisms under multi-source, heterogeneous, and online incremental scenarios remain underdeveloped. Continued advancements are needed to overcome limitations in mitigating negative transfer, improving credibility evaluation, and ensuring physical consistency.

#### 3.2.6. Reinforcement Learning

Reinforcement Learning (RL) is a branch of machine learning in which an algorithm interacts with an environment and seeks to take actions that maximize cumulative rewards. The algorithm, known as an ”agent”, learns by taking actions in the environment and receiving feedback in the form of rewards. The goal of the agent is to learn a policy that selects actions leading to the highest long-term reward. In other words, the agent aims to learn an optimal sequence of actions by observing the outcomes of its interactions with the environment.

While traditional machine learning and deep learning algorithms have demonstrated impressive performance on well-structured datasets, they often exhibit limited adaptability to dynamic environments and struggle to handle previously unseen faults. In recent years, there has been growing interest in applying RL to machinery fault detection, diagnosis, classification, and Remaining Useful Life (RUL) prediction. RL has been successfully employed in various domains, including fault diagnosis of power transmission lines [98], optimization of smart grid operations [99], fault detection in hydraulic presses [100], and industrial process control.

The training mechanism of Reinforcement Learning (RL) facilitates the algorithm’s adaptability to varying environmental conditions, making it particularly suitable for addressing such challenges. Xiaofeng Liu et al. [101] proposed an Environment-Adaptive Deep Reinforcement Learning fault diagnosis method (EA-DRL). The core idea of EA-DRL is to leverage a domain generalization (DG) network to extract both domain-invariant and domain-specific features while employing a Double Dueling Deep Q-Network (D3QN) to enhance self-predictive capabilities in unknown environments. Compared to conventional RL approaches, EA-DRL introduces a Weighted Normalized Mutual Information (WNMI)-based feature selection strategy to eliminate redundancy and improve feature discriminability during agent–environment interaction. Furthermore, a novel reward mechanism based on Environmental Discrimination Reward (EDR) and State Identification Reward (SIR) is designed, enabling the agent to act solely based on fault states, independent of environmental variations. Experimental results show that EA-DRL exhibits strong generalization and robustness in cross-environment fault diagnosis tasks. Xingqiu Li et al. [102] developed a Reinforced Ensemble Deep Transfer Learning Network (REDTLN) to address Transfer Learning problems in multi-source domains. The approach consists of three key components: single-source to single-target domain adaptation, a unified metric design for unsupervised ensemble learning, and a multi-source, multi-model reinforced ensemble mechanism. The method was validated on multiple rolling bearing datasets and demonstrated superior fault pattern recognition accuracy and enhanced robustness. Shuilong He et al. [103] introduced a novel deep Reinforcement Learning strategy that integrates SimCLR and Prioritized Experience Replay (PER) for quantitative fault diagnosis under non-ideal data conditions. The proposed method achieved automatic and accurate qualitative fault identification under varying rotational speeds, loads, and class imbalance scenarios, demonstrating excellent effectiveness, stability, and generalizability.

Compared to traditional deep learning methods that rely on static datasets, Reinforcement Learning (RL) inherently possesses environmental adaptability and the ability to explore unknown faults. As a result, it can maintain high diagnostic accuracy in scenarios involving previously unseen faults, variable operating conditions, and limited data availability. However, RL suffers from low sampling efficiency and often requires extensive interaction to converge in high-dimensional state–action spaces—something real-world industrial systems cannot afford due to the downtime and safety risks associated with frequent trial-and-error learning. Furthermore, RL training processes often lack convergence stability and are highly sensitive to random seeds and hyperparameters, making it difficult to ensure reproducibility in critical industrial applications. These limitations significantly constrain the large-scale deployment of RL in safety-critical and time-sensitive industrial environments.

Deep learning methods exhibit strong feature extraction and fault identification capabilities in industrial fault diagnosis, significantly enhancing diagnostic accuracy and efficiency. However, these approaches still face challenges such as limited generalization and dependence on large amounts of labeled data, particularly in complex industrial settings with diverse fault patterns. To address these constraints, large-model approaches have begun to receive increased attention. The following section explores the application and advantages of large models in industrial fault diagnosis.

### 3.3. Fault Diagnosis Methods Based on Large Model

Large models refer to neural networks with massive numbers of parameters and deep layers, exhibiting powerful learning and generalization capabilities. Since the introduction of ChatGPT [104] in November 2022, Large Language Models (LLMs) have advanced rapidly. Models such as PaLM [105], LLaMA [106], and GPT-4 [107] have been successively released, achieving outstanding performance on diverse natural language tasks. Driven by the notable success of LLMs, the computer vision community has also adopted large-model strategies—for instance, partitioning images into small patches to accommodate large-scale models [108] —resulting in significant improvements in image classification, object detection, and image segmentation.

**Figure 12:**
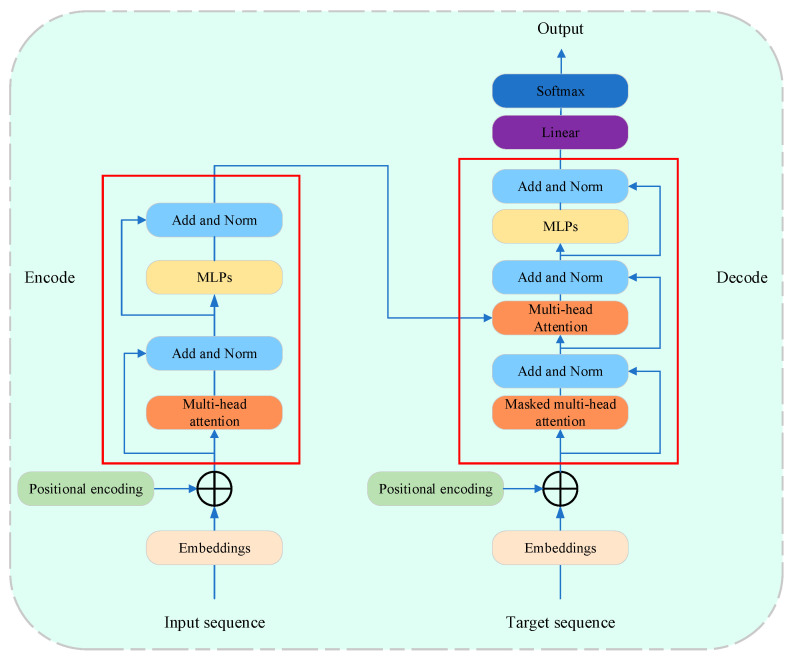
Transformer Structure Diagram.

Currently, the most widely used LLM architectures include encoder-only, decoder-only, and encoder–decoder models, most of which use the Transformer [109] as a foundational block. Initially proposed for machine translation, the Transformer language model architecture is composed of an encoder and a decoder. The encoder consists of *N* = 6 identical Transformer layers, each containing two sub-layers: a multi-head self-attention mechanism and a simple position-wise fully connected feed-forward network. The decoder is also formed by stacking six identical layers; however, in addition to the two sub-layers present in each encoder layer, the decoder has a third sub-layer that performs multi-head attention over the encoder stack’s outputs. The architecture is illustrated in Figure 12 [110].

In the context of industrial fault diagnosis, Laifa Tao et al. [111] proposed a bearing fault diagnosis framework based on LLMs. Their approach consists of three main steps. First, they introduce a signal feature quantization method to address the challenge of extracting semantic information from vibration data, combining time–frequency-domain features derived from a statistical analysis framework. Next, they transform the time-series data into textual form, aiming to learn common features efficiently under cross-conditions and limited data scenarios through succinct feature selection. Finally, they adopt LoRA and QLoRA for fine-tuning, thereby enhancing the LLM’s ability to generalize when analyzing vibration data features. The model’s performance was validated through complete-data and limited-data experiments, covering both single cross-condition and cross-dataset transfers. The results demonstrate the proposed framework’s capability to perform three types of generalization tasks simultaneously, effectively boosting the model’s generalization performance.

One of the goals of intelligent fault diagnosis is to detect anomalies directly from industrial scene images and to describe the anomalies in detail. To this end, Zhaopeng Gu et al. [18] introduced computer vision techniques into large models for industrial fault diagnosis, proposing a novel method known as AnomalyGPT. By analyzing abnormal images and generating corresponding textual descriptions for each image, they create training data. The approach includes an image decoder to provide fine-grained semantic information and a prompt learner for fine-tuning the Large Vision-Language Model (LVLM) via prompt embeddings. AnomalyGPT eliminates the need for manually setting thresholds, thereby enabling direct evaluation of anomaly presence and localization. It also supports multi-round dialogue and exhibits strong contextual learning capabilities.

**Figure 13:**
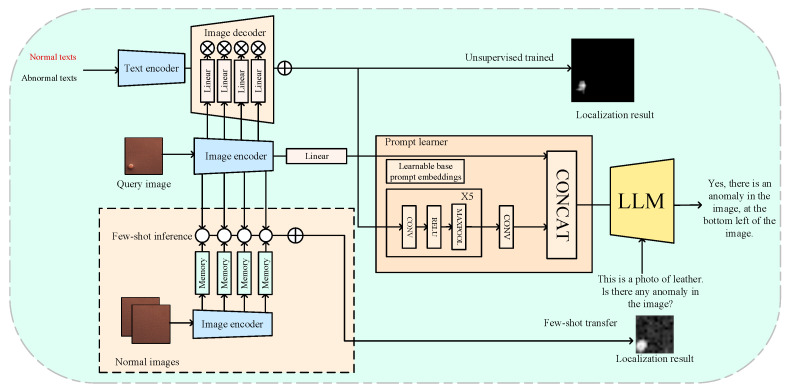
AnomalyGPT structure diagram.

Figure 13 depicts the overall architecture of AnomalyGPT. For an input image $x\in {\mathbb{R}}^{H\times W\times C}$, the image encoder extracts features and passes them through a linear layer to obtain the embedded image $E_{img}\in {\mathbb{R}}^{C_{emb}}$, which is then fed into the LLM. Under an unsupervised setting, block-level features extracted from intermediate encoder layers are combined with text features and passed to the decoder, yielding pixel-level anomaly localization. In a few-shot setting, block-level features derived from normal samples are stored in a memory bank; anomaly localization is then determined by computing the distance between a query block and the most similar block in the memory bank. The localization results are converted into prompt embeddings by the prompt learner, forming part of the LLM’s input. The LLM then uses the image input, prompt embeddings, and user-supplied text input to detect anomalies and identify their positions, ultimately generating a response for the user. On the MVTec-AD dataset, AnomalyGPT achieves state-of-the-art performance with only one normal sample, attaining an accuracy of 86.1%, an Image-level AUC of 94.1%, and a Pixel-level AUC of 95.3%.

In another study, Peifeng Liu et al. [112] proposed a knowledge-enhanced joint model that embeds an aeronautical assembly Knowledge Graph (KG) into an LLM. This model leverages graph-structured big data from KGs to perform prefix tuning on the LLM, allowing online reconfiguration and avoiding excessive computational overhead. In real-world industrial scenarios, the enhanced joint model achieved a 98.5% accuracy rate in both fault diagnosis and troubleshooting.

Furthermore, Yuanze Li et al. [19] presented Myriad, an innovative multimodal large model that delivers precise anomaly detection and high-quality anomaly descriptions by incorporating the prior knowledge of a “vision expert”. Myriad is built on MiniGPT-4 and introduces an expert-awareness module, which transforms the vision expert’s knowledge into tokens interpretable by the LLM. To address potential errors or ambiguities from the vision expert, a domain adapter is included to handle differences in visual representations between general and industrial images. Additionally, a vision-expert guidance module enables Q-Former to generate vision-language tokens specific to fault diagnosis, guided by the vision expert’s prior knowledge. Comprehensive experimental results show that Myriad performs exceptionally well on the MVTec-AD benchmark, particularly in one-class and few-shot settings, offering accurate anomaly predictions and generating detailed anomaly descriptions.

Large-scale models (LLMs) demonstrate significant advantages in handling complex tasks and generating high-quality outputs. They not only provide accurate anomaly localization but also produce detailed fault descriptions, thereby greatly enhancing the level of automation and intelligence in fault diagnosis. However, several challenges still hinder their widespread application in real-world industrial settings. First, inference with models containing billions of parameters typically requires GPU clusters or specialized accelerators, far exceeding the power and spatial budgets of edge devices deployed at the workshop level. Second, during the generative process, LLMs may inadvertently expose sensitive information embedded in training data, such as proprietary processes or personal data, raising compliance concerns under regulations like GDPR and PIPL. Third, fine-tuning for rare or emerging fault patterns still relies on manually labeled data, which is susceptible to class imbalance and prone to overfitting in few-shot learning scenarios.

Looking ahead, as computational costs decline and regulatory–compliant data collaboration and self-supervised cross-modal pretraining paradigms mature, LLM-based industrial anomaly detection is expected to overcome existing performance bottlenecks and emerge as a key enabler in intelligent manufacturing systems. Simultaneously, optimizing model architectures, reducing computational overhead, and improving training efficiency will be critical directions for advancing this domain further and establishing LLMs as indispensable tools in the industrial intelligence landscape.

## 4. Case Analysis

This section focuses on analyzing representative case studies corresponding to the three categories of data-driven fault diagnosis methods summarized in Section 3. These case studies serve as practical references for the application of the aforementioned diagnostic approaches and are categorized based on traditional machine learning, deep learning, and modern large-scale model techniques.

### 4.1. Two-Tank Study Based on Fuzzy C-Means

This case addresses the pervasive issues of uncertainty and fuzziness in industrial data by proposing a hybrid algorithmic strategy: the Interval Type-2 Fuzzy C-Means Algorithm and Its Kernel Alternative (KIT2FCM). This approach enhances the traditional Fuzzy C-Means (FCM) by simultaneously introducing kernel mapping and type-2 membership functions, enabling the model to capture nonlinear decision boundaries and quantify measurement uncertainty more effectively. The proposed method integrates both fault and cyberattack detection and classification, offering a robust solution to the uncertainty in sensor measurements caused by noise and interference in industrial processes. Moreover, the algorithm allows for greater separability between classes, improving overall diagnostic performance in complex industrial environments [113].

The effectiveness of the proposed method was validated using the Two-Tank system [114], a benchmark for complex industrial processes. The diagnostic framework operates in two distinct phases: offline training and online analysis. In the offline phase, the model is trained using historical data, during which parameter calibration and performance baseline evaluation are conducted. Once convergence is achieved, the class centers and thresholds are fixed. In the online phase, the model requires only a one-time calculation of the kernel distances between the current sample and each class center, followed by membership degree estimation, enabling rapid classification without the need for retraining.

To assess the robustness of the proposed approach, the authors conducted three experiments under varying levels of observation noise: (1) noise-free measurements, (2) measurements with 2% noise, and (3) measurements with 5% noise. Experimental results demonstrated that KIT2FCM maintained an average sensitivity of ≥90% and an acceptable false positive rate in the online monitoring of the Two-Tank process, even under 5% noise conditions. These findings validate the capability of the “kernel mapping + interval type-2 fuzzy” mechanism to suppress random disturbances effectively. Detailed results are presented in Table 4.

### 4.2. LSTM-Based Fault Diagnosis in the Tennessee Eastman (TE) Process

To address the classification challenges caused by uncertainty in industrial data and the potential overlap between fault and attack categories, the authors proposed an LSTM-based state monitoring strategy [115]. This approach integrates both fault diagnosis and cyberattack detection/localization within industrial plants and is particularly notable for its robustness against external disturbances and noise.

The strategy leverages the Long Short-Term Memory (LSTM) network’s ability to model temporal dependencies in multivariate time-series data, enabling the system to accurately distinguish between various fault and attack conditions, even in noisy environments. Experimental results demonstrated that the LSTM-based method maintains high performance in terms of both detection accuracy and stability, confirming its suitability for real-world industrial applications where noise and uncertainty are inherent.

The authors validated their approach using the classic “Tennessee Eastman (TE) process”, which consists of 52 process and control variables and includes 21 predefined fault scenarios [116]. From these, they selected three fault types, F3, F9, and F15, that exhibit the greatest overlap with normal operating conditions. Additionally, three stealthy sensor spoofing attacks, A1 to A3, were crafted to evaluate the model’s comprehensive performance under the dual threat of conventional faults and cyberattacks.

The LSTM-based deep learning classifier used in this study features an input layer of dimension 52, followed by three stacked LSTM hidden layers. Dropout layers are inserted between LSTM blocks to mitigate overfitting. The final output is produced through a fully connected layer followed by a Softmax activation, yielding seven classification labels: normal operating condition (NOC), the three fault types, and the three attack types. As shown in Table 5, the model achieved an average classification accuracy of 98.86% across all seven categories. Even in the presence of measurement noise and significant class overlap, the model consistently maintained around 99% overall accuracy, demonstrating its strong adaptability to external disturbances and its robustness in complex industrial environments.

### 4.3. AnomalyGPT Based on LVLM

This section provides an in-depth discussion of the practical application and experimental results of the novel industrial fault diagnosis method AnomalyGPT, based on LVLMs, as previously introduced.

As shown in Table 6, AnomalyGPT effectively satisfies all the current requirements for industrial fault diagnosis, including few-shot learning, anomaly scoring, anomaly localization, anomaly classification, and multi-round dialogue. In the table, “traditional fault diagnosis methods” refer to “one-class-one-model” approaches such as PatchCore [117], InTra [118], and PyramidFlow [119]; “few-shot learning fault diagnosis methods” are those that utilize a small number of samples for learning, such as RegAD [120], Graphcore [121], and WinCLIP [122]; and “LVLMs” represent general Large Vision-Language Models such as MiniGPT-4 [123], LLaVA [124], and PandaGPT [125].

The experiments were conducted on the MVTecA and VisA [126] datasets, with Image-level AUC (Image-AUC) and Pixel-level AUC (Pixel-AUC) used to assess anomaly detection and localization performance. The MVTec-AD dataset consists of 3629 training images and 1725 test images across 15 distinct categories. The training set contains only normal images, while the test set includes both normal and abnormal images. VisA is a newly introduced dataset containing 9621 normal images and 1200 abnormal images across 12 categories.

In the implementation, ImageBind-Huge was used as the image encoder, and Vicuna-7B served as the inference Large Language Model connected through a linear layer. The model was initialized using pre-trained parameters from PandaGPT, and alternating training was performed between the pre-trained data from PandaGPT and the abnormal image-text pairs from the dataset. Only the decoder and prompt learner were fine-tuned, while the remaining parameters were fixed.

For performance comparison, baseline methods such as SPADE [127], PaDiM [128], PatchCore, and WinCLIP were selected and compared with AnomalyGPT. The results presented in Table 7 demonstrate that AnomalyGPT significantly outperforms prior methods in Image-AUC and achieves competitive Pixel-AUC and strong accuracy.

## 5. Current Challenges and Future Outlook

### 5.1. Problem Summary

In response to the numerous challenges identified in current research, scholars have proposed a variety of improvement methods, which, to some extent, reflect common characteristics. Through a systematic discussion of the aforementioned problems and corresponding methods, existing approaches can be broadly categorized into two main types: data-level methods and network-level methods.

Data-level methods primarily focus on enriching and optimizing training data by incorporating advanced signal processing techniques and implementing data augmentation strategies (such as data expansion and multi-source data fusion) to improve the model’s generalization capability. On the other hand, network-level methods concentrate on optimizing the structure design and training process of neural networks, including improving network architectures, developing more effective loss functions, and introducing new network models (such as large models). By utilizing more complex and deeper model structures, network-level methods aim to enhance the model’s learning ability and capacity to capture complex patterns. These methods have been applied across various research problems, demonstrating their broad applicability and importance.

### 5.2. Future Research Trends

#### 5.2.1. Industrial Data Quality

In the era of Intelligent Fault Diagnosis, the scale of data collection is growing at an unprecedented pace. However, the quality of the collected data is not always satisfactory, and some may suffer from poor quality. Low-quality data refer to unreliable data that are inaccurate, uncertain, incomplete, or lacking in timeliness. When such low-quality data are directly used to train machine learning diagnostic models, this can lead to unreliable diagnostic results, undermining the model’s effectiveness and credibility. Therefore, there is an urgent need to develop effective methods for collecting and cleaning incorrect data to enhance the overall quality of the big data collected.

An end-to-end data governance pipeline covering the entire process, from data acquisition → preprocessing → storage → consumption, can be established to enhance diagnostic reliability. Quality should be assessed using quantitative metrics such as consistency, completeness, accuracy, and timeliness, which form the foundation for reliable fault diagnosis. At the same time, ”federated active learning” is emerging as a viable approach for improving low-quality data. In this framework, the model locally selects the most informative samples based on uncertainty or diversity criteria, and these samples are then labeled or corrected by field engineers. The global model parameters are aggregated through privacy-preserving mechanisms, significantly improving fault recognition accuracy without exposing raw source data.

In the future, “open benchmarking standards and competitions” will provide a unified platform to compare different data cleaning and enhancement strategies, accelerating the implementation and iteration of data quality management techniques.

#### 5.2.2. Interpretability of Deep Learning

Diagnostic models based on deep learning are typically constructed through repeated experimental iterations rather than being grounded in a strict theoretical framework. The process of learning model parameters often relies on good initial values rather than strictly derived results. This experimental dependency leads to a certain lack of theoretical interpretability in the model. The interpretability of a model is crucial for users to understand how the diagnostic model learns and extracts useful fault information from sensor data. A significant limitation of deep learning in Intelligent Fault Diagnosis applications is that the method often operates like a “black box”, making it difficult to explain the internal workings of the model and to gain insights into how and why it makes final decisions. This “black box” effect makes it difficult to theoretically explain and verify many key issues, limiting the credibility and reliability of the model in practical applications.

Recent reviews have shown that combining local gradient-based visualization methods (such as Grad-CAM and Integrated Gradients) with feature-level interpreters (such as SHAP and LIME) can effectively highlight the key regions or frequency bands of model attention across both image and time-series domains. This dual-scale interpretability significantly enhances engineers’ trust in the diagnostic outcomes. Furthermore, by leveraging Physics-Informed Neural Networks (PINNs), physical mechanisms of fault propagation or energy conservation equations can be explicitly embedded into the loss function. This approach preserves the expressive power of deep learning models while simultaneously outputting physically verifiable intermediate quantities, thereby achieving a balance between prediction accuracy and interpretability.

In industrial applications, particularly in fault diagnosis of critical components, there are high demands for accuracy and rigor. Significant fluctuations in diagnostic accuracy are unacceptable, which contrasts sharply with fields where accuracy requirements are relatively lower. Therefore, enhancing the interpretability of deep learning models not only helps improve their reliability and user trust but is also key to ensuring that deep learning plays a vital role in industrial scenarios.

#### 5.2.3. Edge Large Models

LLMs based on Transformers have made significant progress, and their deployment on resource-constrained edge devices in the industrial domain is rapidly gaining traction. This approach is known as Edge-based Large Language Models [129], where LLMs are deployed directly on edge devices instead of relying on centralized cloud servers.

In the field of industrial fault diagnosis, this approach offers significant advantages. First, edge computing enables LLMs to operate locally on the device, providing faster response times and low-latency analysis, along with the ability to execute maintenance actions directly. Second, local execution allows LLMs to function without an internet connection, which is particularly important in industrial environments with limited network connectivity, such as remote mining sites, offshore platforms, or underground facilities. Finally, deploying LLMs on edge devices enhances privacy and security by processing sensitive industrial data locally. Industrial data often contain trade secrets and core technical information, so keeping these data on the device prevents leakage of critical information.

Recent studies have demonstrated that 4-bit mixed-precision quantization, when combined with structured pruning and low-rank adaptation techniques (e.g., LoRA/QLoRA), can reduce the size of Large Language Models (LLMs) by 80–90% and lower inference latency by 3–5 times, all while maintaining accuracy loss within 1%. At the hardware–algorithm co-design level, techniques such as streaming weight loading, Flash-Attention, and on-chip SRAM pipeline parallelism can fully exploit the throughput potential of ARM NPUs and RISC-V accelerators. For nodes with more constrained computational resources, a hierarchical inference architecture of “lightweight edge frontend + cloud expert model” can be employed. In this setup, the edge device performs rapid anomaly screening while complex semantic analysis and model updates are handled in the cloud. An adaptive task offloading strategy enables dynamic switching between edge and cloud processing to optimize performance. Therefore, deploying LLMs at the edge can improve the timeliness, efficiency, and security of industrial fault diagnosis, driving industrial systems toward greater intelligence and autonomy.

## 6. Conclusions

This review charts the progression of industrial fault diagnosis from rule-based reasoning and classical signal-processing techniques to contemporary data-driven paradigms powered by industrial big data ecosystems, deep neural networks, and, most recently, Large Vision-Language Models. We first clarified the provenance, characteristics, and management platforms of industrial big data, demonstrating how the escalating volume, variety, and velocity of multi-source heterogeneous signals have reshaped diagnostic workflows and motivated the development of public benchmark datasets. Next, we compared three successive generations of data-driven approaches—traditional machine learning, deep learning, and large-model frameworks—detailing their fundamental principles, strengths, and limitations and illustrating each with representative case studies. Finally, we distilled the principal technical challenges into three interrelated research frontiers: systematic data-quality governance, interpretable deep architectures, and resource-efficient edge deployment of large models.

In summary, data-driven fault diagnosis is entering a new phase in which pervasive sensing, foundation-model technologies, and edge intelligence converge. The systematic synthesis and critical insights presented here aim to serve as a valuable guide for researchers and practitioners dedicated to developing the next generation of reliable, transparent, and autonomous industrial maintenance systems.

## Figures and Tables

**Table 1:** Partial list of publicly available fault diagnosis datasets.

Dataset Name	Characteristics	Collection Object	Fault Type	Limitations
CWRU [7]	Includes multiple load conditions, fault severity levels, and fault types. Suitable for various benchmarking experiments and algorithm validation.	Bearings	Inner ring fault, outer ring fault, rolling element fault	Single speed and laboratory artificial defects; single point vibration channel only
IMS [8]	Contains three bearings that transitioned from normal operation to failure, suitable for research on Remaining Useful Life prediction and early fault detection.	Bearings	Inner ring fault, outer ring fault, rolling element fault	Fixed 2000 rpm; terabytes of data; high processing cost
PU [9]	Contains 32 sets of electrical and vibration signals, covering both artificially induced and real faults. Suitable for multi-sensor data fusion research.	Bearings	Artificial inner and outer ring faults, real outer ring faults	Narrow load range and few rolling element samples; still idealistic
SEU [10]	Includes datasets of bearings and gears under different speeds and load conditions. Suitable for studying condition transfer and non-repetitive fault diagnosis.	Gears	Tooth wear, tooth breakage, root crack, surface fault	Most of the gears are wire cutting defects; lack of multimodal signal
MFPT [11]	Includes bearing data collected under different load conditions. Suitable for research on condition transfer and the impact of load variation on fault diagnosis.	Bearings	Inner ring fault, outer ring fault	Large file with high sampling rate; no compound fault
MVTec AD [12]	Contains multiple types of high-quality normal and abnormal samples. Applicable for surface defect monitoring and anomaly detection in industrial products.	Various industrial products	Surface defects, structural anomalies, etc.	Static visual samples only; lack of operating conditions and fine labels

**Table 2:** Data and diagnostic methods at different stages.

Stage	Data Characteristics	Diagnosis Method	Advantages	Limitations
Early Diagnosis Stage	Small data volume, single data type	Principle-based reasoning, expert knowledge	Simple and direct approach	Limited accuracy and reliability
Sensor Stage	Medium-scale data, multi-type signals	Signal processing, analytical models	Capable of processing multiple signals, extracting general patterns	Challenging to process high-dimensional, multi-source data
Big Data Stage	Massive data, multi-modal signals, heterogeneous data fusion	Machine learning, deep learning, pre-trained large models	Automatically extracts deep features, strong generalization capability	Requires extensive labeled data, high model complexity, challenges in interpretability and transparency

**Table 3:** Comparison summary table of three types of methods.

Method Category	Typical Techniques/Algorithms	Main Advantages	Main Limitations	Suitable Scenarios
Knowledge-based	Expert systems, fault tree analysis (FTA)	Transparent, fully explainable rules; highly targeted for specific equipment	Knowledge elicitation and updates are time-consuming; limited coverage of novel or complex faults	Plants with ample expert know-how and stable operating conditions; applications that require auditable, rule-based diagnosis
Signal- and model-based	Spectrum/envelope analysis, wavelet transform, finite element/data assimilation models	Strong early-warning capability; real-time monitoring feasible	Dependence on accurate physical models and high-quality signals; sensitive to multivariate coupling and noise	Machinery whose physics are well understood and whose fault signatures are distinctive (e.g., rotating machines, structural components)
Data-driven	Machine learning (SVM, random forest), deep learning (CNN/LSTM), large models (LLM/VLM)	Handles high-dimensional, nonlinear data; cross-condition adaptability; end-to-end learning	Requires large volumes of labeled data and significant compute; limited interpretability; vulnerable to data-shift issues	Modern smart factories with rich sensor networks, large data volumes, and highly variable operating conditions

**Table 4:** Results in % of the sensitivity and (1-specificity).

Scenario	Without Noise		2% Noise Level		5% Noise Level	
	Sensitivity	1-Specificity	Sensitivity	1-Specificity	Sensitivity	1-Specificity
F1	96	0	94	4.08	92	6.12
A1	92	9.8	88	13.63	86	17.31
F2	100	0	96	2.04	94	6
A2	94	0	92	2.13	90	4.26

**Table 5:** Confusion matrix and per-class accuracy of LSTM classifier.

	NOC	F3	F9	F15	A1	A2	A3	TA (%)
**NOC**	500	0	0	0	0	0	0	100
**F3**	0	500	0	0	0	0	0	100
**F9**	7	0	488	5	0	0	0	97.6
**F15**	10	2	0	488	0	0	0	97.6
**A1**	5	3	2	0	490	0	0	98
**A2**	4	0	2	0	0	494	0	98.8
**A3**	0	0	0	0	0	0	500	100
**AVE**								98.86

**Table 6:** Comparison of AnomalyGPT with existing methods in practical applications.

Methods	Few-Shot Learning	Anomaly Score	Anomaly Localization	Anomaly Judgement	Multi-Turn Dialogue
Traditional IAD methods		√	√		
Few-shot IAD methods	√	√	√		
LVLMs	√				√
AnomalyGPT	√	√	√	√	√

**Table 7:** Experimental results of AnomalyGPT and other methods on small-sample data.

Setup	Method	MVTec-AD			VisA		
		Image-AUC	Pixel-AUC	Acc	Image-AUC	Pixel-AUC	Acc
l-shot	SPADE	81.0 ± 2.0	91.2 ± 0.4	-	79.5 ± 4.0	95.6 ± 0.4	-
	PaDiM	76.6 ± 3.1	89.3 ± 0.9	-	62.8 ± 5.4	89.9 ± 0.8	-
	PatchCore	83.4 ± 3.0	92.0 ± 1.0	-	79.9 ± 2.9	95.4 ± 0.6	-
	WinCLIP	93.1 ± 2.0	95.2 ± 0.5	-	83.8 ± 4.0	96.4 ± 0.4	-
	AnomlyGPT	94.1 ± 1.1	95.3 ± 0.1	86.1 ± 1.1	87.4 ± 0.8	96.2 ± 0.1	77.4 ± 1.0
2-shot	SPADE	82.9 ± 2.6	92.0 ± 0.3	-	80.7 ± 5.0	96.2 ± 0.4	-
	PaDiM	78.9 ± 3.1	91.3 ± 0.7	-	67.4 ± 5.1	92.0 ± 0.7	-
	PatchCore	86.3 ± 3.3	93.3 ± 0.6	-	81.6 ± 4.0	96.1 ± 0.5	-
	WinCLIP	94.4 ± 1.3	96.0 ± 0.3	-	84.6 ± 2.4	96.8 ± 0.3	-
	AnomlyGPT	95.5 ± 0.8	95.6 ± 0.2	84.8 ± 0.8	88.6 ± 0.7	96.4 ± 0.1	77.5 ± 0.3
4-shot	SPADE	84.8 ± 2.5	92.7 ± 0.3	-	81.7 ± 3.4	96.6 ± 0.3	-
	PaDiM	80.4 ± 2.5	92.6 ± 0.7	-	72.8 ± 2.9	93.2 ± 0.5	-
	PatchCore	88.8 ± 2.6	94.3 ± 0.5	-	85.3 ± 2.1	96.8 ± 0.3	-
	WinCLIP	95.2 ± 1.3	96.2 ± 0.3	-	87.3 ± 1.8	97.2 ± 0.2	-
	AnomlyGPT	96.3 ± 0.3	96.2 ± 0.1	85.0 ± 0.3	90.6 ± 0.7	96.7 ± 0.1	77.7 ± 0.4

## Abbreviations

The following abbreviations are used in this manuscript:

**Abbreviations**	**Description**
GE	General Electric
ERP	Enterprise Resource Planning
SCM	Supply Chain Management
CRM	Customer Relationship Management
WRA	Water Research Association
SVM	Support Vector Machine
PSO	Particle Swarm Optimization
MFCC	Mel-Frequency Cepstral Coefficients
GTCC	Gammatone Cepstral Coefficients
OC-SVM	One-Class Support Vector Machine
SVR	Support Vector Regression
KNN	K-Nearest Neighbors
PGM	probabilistic graphical models
DBNs	Deep Belief Networks
CNNs	Convolutional Neural Networks
RNNs	Recurrent Neural Networks
LSTM	Long Short-Term Memory
GANs	Generative Adversarial Networks
RBMs	Restricted Boltzmann Machines
BP	BackPropagation
IMFs	Intrinsic Mode Functions
EEMD	Ensemble Empirical Mode Decomposition
STF-DBN	spatio-temporal features fusion based on deep belief network
PCA	Principal Component Analysis
SAE-DBN	Sparse Autoencoder–Deep Belief Network
DAE	Denoising Autoencoder
TCN	Temporal Convolutional Network
SVDD	Support Vector Data Description
HSFT	Hilbert Spectrum Fusion Technology
MSCNN	Multi-Scale CNN
TICNN	Training-Interference CNN
ECNN	Enhanced CNN
GRU-NP-DAE	GRU-based Nonlinear Prediction Denoising Autoencoder
DCGAN	Deep Convolutional GAN
CGAN	Conditional GAN
ACGAN	Auxiliary Classifier GAN
HVAC	heating, ventilation, and air conditioning
MoGAN	Minority Over-sampling GAN
WGAN	Wasserstein GAN
LLMs	Large Language Models
LVLM	Large Vision-Language Model
KG	Knowledge Graph
Image-AUC	Image-level AUC
Pixel-AUC	Pixel-level AUC
TL	Transfer Learning
DTL	Deep Transfer Learning
RL	Reinforcement Learning
SD	source domain
TD	target domain
RUL	Remaining Useful Life
EA-DRL	Environment-Adaptive Deep Reinforcement Learning
D3QN	Double Dueling Deep Q-Network
WNMI	Weighted Normalized Mutual Information
EDR	Environmental Discrimination Reward
SIR	State Identification Reward
REDTLN	Reinforced Ensemble Deep Transfer Learning Network
PER	Prioritized Experience Replay
FCM	Fuzzy C-Means
TE	Tennessee Eastman process
